# Exploration of avocado by-products as natural sources of bioactive compounds

**DOI:** 10.1371/journal.pone.0192577

**Published:** 2018-02-14

**Authors:** Maria Augusta Tremocoldi, Pedro Luiz Rosalen, Marcelo Franchin, Adna Prado Massarioli, Carina Denny, Érica Regina Daiuto, Jonas Augusto Rizzato Paschoal, Priscilla Siqueira Melo, Severino Matias de Alencar

**Affiliations:** 1 Department of Agri-food Industry, Food and Nutrition, "Luiz de Queiroz" College of Agriculture, University of São Paulo, Piracicaba, SP, Brazil; 2 Piracicaba Dental School, Department of Physiological Sciences, University of Campinas, Piracicaba, SP, Brazil; 3 School of Agricultural Sciences, State University Paulista “Júlio de Mesquita Filho”, Rua José Barbosa de Barros, Botucatu, SP, Brazil; 4 Department of Physics and Chemistry, School of Pharmaceutical Sciences of Ribeirão Preto, University of São Paulo, Vila Monte Alegre, Ribeirão Preto, SP, Brazil; College of Agricultural Sciences, UNITED STATES

## Abstract

This study aimed to evaluate the antioxidant, anti-inflammatory, and cytotoxic properties and phenolic composition of peel and seed of avocado varieties Hass and Fuerte using green solvents. Ethanol soluble compounds were identified in peel and seed of both varieties using HPLC-MS/MS and quantified using HPLC-DAD. Agro-industrial by-products of both varieties exhibited high radical scavenging activity against synthetic free radicals (DPPH and ABTS) and reactive oxygen species (peroxyl, superoxide, and hypochlorous acid) and high ability to reduce Fe^3+^ to Fe^2+^. The main compounds with significant contribution to the antioxidant activity determined by online HPLC-ABTS^●+^ analyses were procyanidin B_2_ and epicatechin in the peel and trans-5-*O-*caffeoyl-D-quinic acid, procyanidin B_1_, catechin, and epicatechin in the seed. Peel of Fuerte significantly suppressed TNF-α and nitric oxide (NO) release (459.3 pg/mL and 8.5 μM, respectively), possibly because of the high phenolic content and antioxidant activity detected. Avocado agro-industrial by-products can be used for food and pharmaceutical purposes due to their antioxidant and anti-inflammatory properties.

## Introduction

Avocado (*Persea Americana* Mill., Lauraceae) is an important fruit native to Central America and Mexico and cultivated in almost all tropical and subtropical regions worldwide. The world production of avocado was around five million tons in 2014. The largest producer of avocado is Mexico, country that accounts for 30% of the world production. Brazil produced 157,000 tons in 2014 [1, 2].

Avocado fruits have high nutritional quality and contain high levels of vitamins, minerals, proteins, and fibers, as well as high concentrations of unsaturated fatty acids, beneficial to health [3]. In addition, avocado peel and seed have high contents of bioactive phytochemicals such as phenolic acids, condensed tannins, and flavonoids, including procyanidins, flavonols, hydroxybenzoic, and hydroxycinnamic acids [4–6]. These bioactive compounds have shown various biological activities such as antioxidant and anti-inflammatory properties. The anti-inflammatory activity of phenolic compounds is largely related to their ability to scavenge oxidative radicals, which is important for cell and oxidative stress regulation [7].

Different varieties of avocado are cultivated worldwide, and Hass and Fuerte are among the most consumed ones [2]. Avocado industrial processing generates large quantities of agro-industrial by-products, mainly peel and seed, ranging from 18% to 23% of fruit dry weight [8]. Thus, it is interesting to reuse these by-products both to reduce their negative impact on the environment and to add value to them, inasmuch as they are sources of important phytochemical compounds [9, 10]. Therefore, the transformation of these agro-industrial by-products, which yield a range of bioactive substances, should be made by green extraction to reduce the environmental impact of the agro-industrial chain.

Green extraction is based on the discovery and design of extraction processes which reduce energy consumption, allow the use of alternative solvents as well as renewable natural products, and ensure the obtention of safe and high quality extracts. Ethanol is the most common bio-solvent and plays an important role in the replacement of petrochemical solvents. Because of its low polarity and very high solvent power, ethanol can be used for the extraction of phenolic compounds from agro-industrial by-products [9, 11].

Therefore, the goal of the present study was to assess the antioxidant and anti-inflammatory properties and bioactive phenolic composition of Hass and Fuerte avocado peel and seed extracts obtained by green extraction. Hass and Fuerte were selected for the present study due to their high economic importance, since they are among the most produced and consumed varieties in the world. For the first time, real-time analyses of phenolic compounds obtained by green extraction of avocado agro-industrial by-products have been carried out using high performance liquid chromatography with diode array detector (HPLC-DAD) and 2,2′-azinobis-(3-ethylbenzothiazoline-6-sulfonic acid) (ABTS) assay, in order to identify their contribution to total bioactivity of avocado by-products.

## Material and methods

### Chemicals

The following chemicals were used in this study: Folin-Ciocalteu reagent (Dinâmica Química Contemporânea, Diadema, SP, Brazil); sodium carbonate, potassium chloride, ethanol (Synth, Diadema, SP, Brazil); monobasic and dibasic potassium phosphate, the standard (±)-6-hydroxy-2,5,7,8-tetramethylchromane-2-carboxylic acid (Trolox), the reagents 1,1-diphenyl-2-pycrylhydrazyl (DPPH), 2,2′-azino-bis (3-ethylbenzothiazoline-6-sulfonic acid) diammonium salt (ABTS), 2,4,6-tripyridyl-s-triazine (TPTZ), potassium peroxydisulfate, fluorescein sodium salt, 2,2′-azobis(2-methylpropionamidine) dihydrochloride (AAPH), β-nicotinamide adenine dinucleotide (NADH), phenazine methosulfate (PMS), nitrotetrazolium blue chloride (NBT), sodium hypochlorite solution (NaOCl), rhodamine 123, sodium acetate trihydrate, 3-(4,5-dimethylthiazol-2-yl)-2,5-diphenyltetrazolium bromide (MTT), *Escherichia coli* lipopolysaccharides (LPS), Griess reagent, the authentic standards of flavonoids, and the phenolic acids caffeic acid, catechin, cinnamic acid, epicatechin, epicatechin-3-*O*-gallate, ferulic acid, gallic acid, *p*-coumaric acid, procyanidin B_1_, procyanidin B_2_, and quercetin were purchased from Sigma-Aldrich (St. Louis, MO, USA); acetonitrile and methanol were purchased from J. T. Baker (Phillipsburg, NJ, USA); formic acid was purchased from Tedia (Fairfield, OH, USA); fetal bovine serum (FBS), antibiotics (streptomycin/penicillin), and Dulbecco’s modified Eagle medium (DMEM) were purchased from Vitrocell Embriolife (Campinas, SP, Brazil); water was obtained from a Millipore Milli-Q System (Millipore SAS, Molsheim, France).

### Extraction of avocado agro-industrial by-products

Avocado fruits of varieties Hass and Fuerte were provided by Jaguacy Avocado Brasil, located in the municipality of Bauru, SP, Brazil (22°19’18”S, 49°04’13”W, at 526 m elevation), harvested in June 2011 and April 2012, respectively. The pulp was separated manually from the peel and seed. These agro-industrial by-products were frozen, lyophilized, ground, and stored at -18°C until the extraction assays. The peel and seed were separately analyzed as follows: each material was weighed (1 g) and an aliquot of 10 mL of the solvent (ethanol/water, 80/20 v/v) was added. These extracts were sonicated in an ultrasonic bath Unique of 2.8 L (model USC 1400A, 40 kHz of ultrasound frequency, 135 W RMS power, Indaiatuba, SP, Brazil) for 15 min at room temperature (25 ^o^C) and centrifuged using an Eppendorf 5810R centrifuge (Eppendorf AG, Hamburg, Germany) at 5000 *g* for 15 min. The supernatant was filtered and analyzed for the antioxidant activity and phenolic composition. All the extractions were carried out in triplicate.

### Total phenolic content

Total phenolic content was analyzed using the Folin-Ciocalteu method [12]. The calibration curve was constructed using gallic acid at concentrations ranging from 5 to 80 μg/mL.

### Antioxidant activity

The measurement of free radical scavenging activity using DPPH was an adaptation of a method previously used by our team [13]. The calibration curve was built using Trolox at concentrations ranging from 0 to 200 μM.

The determination of the antioxidant capacity by ABTS free radical scavenging was an adaptation of a method previously cited [13]. Trolox was used as reference at concentrations ranging from 100 to 2000 μM. Both in DPPH and ABTS methods the results were expressed as μmol Trolox equivalents per g of sample.

The antioxidant capacity was determined using ferric reducing ability of plasma (FRAP) [13]. The calibration curve was constructed with ferrous sulfate at concentrations ranging from 500 to 2000 μM. The results were expressed as μmol Fe^2+^ per g of sample.

The capacity of the avocado peel and seed extracts to deactivate ROS was assessed using three different reactive species: peroxyl, superoxide, and hypochlorous acid. Peroxyl radical (ROO∙) scavenging capacity was measured using Trolox as standard at concentrations ranging from 12.5 to 400 μM, and the results were expressed as Trolox equivalents [9]. The capacity of the extracts to scavenge the superoxide radical (O_2_∙^-^) generated by the NADH/PMS system was also measured [9]. The results were expressed as EC_50_, the mean quantity (mg/mL) of the sample required to quench half of the superoxide radical. Hypochlorous acid (HOCl) scavenging capacity was determined based on the effect of the avocado peel and seed extracts on HOCl-induced oxidation of dihydrorhodamine 123 (DHR) to rhodamine 123 [9]. The results were expressed as EC_50_ (mg/mL) of the sample.

### Determination of phenolic compounds and quantification of free radical scavengers using HPLC-DAD coupled online with ABTS∙^+^ analysis

HPLC-DAD coupled online with ABTS∙^+^ analysis followed an adaptation of a method previously used by our team [14]. The detection of HPLC-separated analytes was performed using DAD, followed by post-column reaction coil using ABTS∙^+^ formed previously, and finally, the detection of the induced bleaching was carried out as a negative peak at 734 nm in an ultraviolet (UV)-Vis detector (SPD-20AV, Shimadzu). We examined the following authentic standards of flavonoids and phenolic acids: caffeic acid, catechin, cinnamic acid, epicatechin, epicatechin-3-*O*-gallate, ferulic acid, gallic acid, *p*-coumaric acid, procyanidin B_1_, procyanidin B_2_, and quercetin. In this study, the detection and quantification limits (μg/mL) were, respectively: caffeic acid– 0.00001 and 0.0003; catechin– 0.025 and 0.0985; epicatechin– 0.0231 and 0.0701; procyanidin B_1_−0.0031 and 0.0095; and procyanidin B_2_−0.0409 and 0.1240.

### Liquid chromatography tandem-mass spectrometry (HPLC-MS/MS) analysis

A Shimadzu HPLC system, composed of an LC 10AD solvent pump, an SLC 10A system controller, and a CTO 10AS column oven, was employed. A Quattro LC triple quadrupole tandem MS/MS system (Micromass, Manchester, UK), with a Z-electrospray interface, set in the negative ion mode, confirmed the identities of the analytes. The collision gas was argon, and nitrogen was employed as desolvation (≅ 380 L/h) and nebulizer (≅ 38 L/ h) gas. During the analyses, the cone, capillary, and extractor were set at 20 V, 3 kV, and 3 V for the electrospray ionization source, respectively. For the confirmation of analyte identities, the multiple-reaction monitoring mode (collision energy of 15 eV) was employed in the analyses.

### Cytotoxicity and anti-inflammatory activity

#### Cell culture

Mouse macrophage RAW 264.7 cells from the American Type Culture Collection (ATCC; Rockville, MD) were cultured in DMEM supplemented with 10% FBS, 100 U/mL penicillin and 100 μg/mL streptomycin at 37°C in 5% CO_2_/95% air.

#### Extract preparation

Avocado peel and seed extracts were prepared as described previously. However, before cytotoxicity and anti-inflammatory assays, the supernatants were lyophilized and diluted at tested concentrations.

#### Cell viability

Cell viability was assessed using a modified MTT assay. Briefly, cells (1 × 10^5^ cells/ well) were seeded in a 96-well plate and incubated at 37°C in 5% CO_2_/95% air. After 24 h, the extracts were added at 0.1, 0.5, 1, 5, 10, 50, and 100 μg/mL concentrations. After 48 h, 100 μL of MTT solution (3 mg/mL in DMEM) was added to each well and further incubated for 3 h at 37°C in 5% CO_2_/95% air. The optical density (OD) of each well was measured at 540 nm in a microplate reader (ASYS, UVM340, Biochrom, UK).

#### Determination of TNF-α production

After 30 min of exposure of the cell culture to 10 μg/mL of the samples, the cells were stimulated with *E*. *coli* LPS (1 μg LPS/mL) and incubated at 37°C for 4 h in humidified 5% CO_2_/95% air. TNF-α was quantified by ELISA on culture supernatant of RAW 264.7 cells. OD readings were performed in a microplate reader (ASYS, UVM340, Biochrom, UK) at 450 nm and the results were expressed in pg/mL.

#### Determination of nitric oxide (NO) production

NO production was determined by measuring the accumulated level of nitrite, an indicator of NO, in the supernatant. After pre-incubation of cells (1 × 10^5^ cells) for 24 h, the extracts (10 μg/mL) were added to LPS (1 μg/mL). The amounts of nitrite, a stable metabolite of NO, were measured using Griess reagent [15].

#### Statistical analysis

The analyses of antioxidant activity were carried out in triplicate. Results of anti-inflamatory assays are mean values of duplicate determinations from three independent experiments. Results are expressed as means ± standard deviation (SD). Data were analyzed using the Tukey’s test (*p* < 0.05) employing the Statistical Analysis System 2002 software (SAS Institute Inc., Cary, NC, USA).

## Results and discussion

### Total phenolic content

To determine the patterns of biologically active compounds accumulation in agro-industrial by-products, it is important to identify their composition and content in separate parts. Ultrasonic extraction was very effective since it decreased the processing time, reduced the cost of extraction, prevented thermal damage, and enhanced the amount of bioactive compounds [16, 17]. Thus, the avocado peel and seed extracts were obtained using this technique and a solution containing ethanol/water (80/20, v/v), because this is a favorable solvent in the extraction of polar substances such as phenolic compounds, does not have toxic effects on humans, and is environmentally friendly [18].

Peel extracts of both avocado varieties tested, Hass and Fuerte, exhibited higher phenolic content (63.5 and 120.3 mg/g, respectively) than seed extracts (57.3 and 59.2 mg/ g, respectively) (Table 1). These results demonstrate that the content of phenolic compounds in our study was significantly higher than that of Hass pulp (0.049 mg/g) [8]. In fact, studies have shown that higher phenolic contents are common in fruit peel and seed [2, 10]. Since ethanol Hass and Fuerte peel and seed extracts displayed high content of phenolic compounds, making them interesting to food and pharmaceutical industries, this work provides new knowledge about total phenolic content in avocado agro-industrial by-products. Therefore, it is valuable to support a search for promising biologically active substances that accumulate in fruit peel and seeds.

**Table 1:** Total phenolic content and antioxidant activity (DPPH, ABTS, FRAP and ROS) of peel and seed extracts of avocado varieties Hass and Fuerte.

Avocado agro-industrial by-products and standards	Total phenolic content (mg/g)^1^	Antioxidant activity					
		DPPH (μmol/g)^2^	ABTS (μmol/g)^2^	FRAP (μmol Fe^2+^/g)^3^	ROS		
					Peroxyl radical (ROO∙) (μmol/g)^2^	Superoxide radical (O_2_∙) (μg/mL)^4^	Hypochlorous acid (HOCl) (μg/mL)^4^
Hass peel	63.5±7.2^B^	310±36.9^D^	791.5± 35.9^B^	1,175.1±102.9^B^	2.8±0.4^C^	52±5^B^	5.2±0.2^C^
Hass seed	57.3±2.7^C^	410.7±35.8^C^	645.8± 17.9^C^	656.9±26.0^D^	2.2±0.7^D^	70 ±2^A^	6.7±0.1^B^
Fuerte peel	120.3±7.8^A^	420.5±23.2^B^	1,004.5±52.0^A^	1,881.4±75.3^A^	9.9±0.9^B^	12 ±3^D^	7.3±0.1^B^
Fuerte seed	59.2±6.9^C^	464.9±32.7^A^	580.8±31.0^D^	931.7±65.6^C^	10.6±1.0^A^	41 ±1^C^	8.6±0.4^A^
Procyanidin B_1_	–^5^	–	–	–	29.2±1.0	–	0.62±0.1
Procyanidin B_2_	–	–	–	–	35.4±1.1	–	0.17±0.03
Catechin	–	–	–	–	24.4±9.6	90 ±1	0.50±0.05
Epicatechin	–	–	–	–	32.2±1.7	227 ±9	0.81±0.01

^1^ Expressed as mg gallic acid equivalents per g of avocado peel and seed (lyophilized).

^2^ Expressed as μmol Trolox equivalents per g of avocado peel and seed (lyophilized).

^3^ Expressed as μmol Fe^2+^ per g of avocado peel and seed (lyophilized).

^4^ EC_50_, i.e., quantity (μg/mL) of avocado peel and seed extract needed to decrease by 50% the amount of the reactive species in the assay.

^5^–: not determined.

DPPH: 1,1-diphenyl-2-pycrylhydrazyl; ABTS: 2,2′-azinobis-(3-ethylbenzothiazoline-6-sulfonic acid); FRAP: ferric reducing ability of plasma; ROS: reactive oxygen species

Values are the means of three replicates ± standard deviation.

Means followed by different capital letters (A, B, C, D) in the same column are significantly different from each other by the Tukey’s test (*p* < 0.05).

### Scavenging of free synthetic radicals, FRAP and ROS

Analyses of synthetic DPPH free-radical scavenging showed that all the peel and seed extracts tested displayed antioxidant activity (Table 1). The antioxidant activity of Fuerte seed extract (464.9 μmol/g) was significantly different from that of peel extract (420.5 μmol/g). High DPPH reducing power has been previously observed in seeds and peel of avocado [8]. Regarding ABTS radical, both varieties tested displayed higher antioxidant capacity in peel extracts (791.5 and 1,004.5 μmol/g, respectively) than in seed extracts (645.8 and 580.8 μmol/g, respectively) (Table 1), a result also reported by other authors [19]. Additionally, higher antioxidant activity has been previously reported for peel compared to seeds of pomegranate (*Punica granatum* L.), rambutan (*Nephelium lappaceum* L.), and mangosteen (*Garcinia mangostana* L.) [20].

Antioxidant activity analyses using FRAP revealed that peel extracts of both varieties reduced twice as much Fe^3+^ into Fe^2+^ compared to seed extracts. The antioxidant activity of Hass and Fuerte peel extracts was 1,175.1 μmol Fe^2+^/g and 1,881.4 μmol Fe^2+^/g, respectively.

Endogenous production of ROS is known to be involved in important physiological functions in different organisms during inflammatory and defense responses against microorganism invasion [21]. However, excessive ROS production generates a redox imbalance, which may cause damage to tissues and/or cells [22].

ORAC has been considered an important method for measuring the capacity of natural compounds to inhibit oxidation reactions by peroxyl free radicals in biological systems [23]. Fuerte peel and seed extracts exhibited higher ROO∙ scavenging capacity (9.9 and 10.6 μmol/g, respectively) than those of Hass (2.8 and 2.2 μmol/g, respectively). Nevertheless, all the avocado extracts tested displayed lower scavenging capacity than procyanidins B_1_ (29.2 μmol/g) and B_2_ (35.4 μmol/g), catechin (24.4 μmol/ g), and epicatechin (32.2 μmol/g) standards at the same concentrations (Table 1).

In both varieties tested, peel extracts displayed higher superoxide radical scavenging capacity (52 μg/mL in Hass and 12 μg/mL in Fuerte) than seed extracts, comparable to that observed for rutin (60 μg/mL), gallic acid (27 μg/mL) [24], quercetin (14 μg/mL), and pulp extracts of strawberry guava (*Psidium cattleianum* Sabine) (20 μg/mL) [25]. Moreover, peel extracts of both varieties exhibited higher superoxide radical scavenging capacity than catechin (90 μg/mL) and epicatechin (227 μg/mL) standards, used as references in this study (Table 1). Superoxide radical (O_2_∙^-^) is constantly produced as a result of several cell processes (mitochondrial and microsomal electron transport chains or enzymes such as xanthine oxidase and NADPH oxidase) or one-electron reduction of O_2_ [21]. After that, it is converted into hydrogen peroxide (H_2_O_2_) by superoxide dismutase, and into hydroxyl radicals (∙OH) via the Fenton or Haber-Weiss reactions [22].

Peel extracts of both avocado varieties also displayed higher HOCl scavenging capacity (5.2 μg/mL in Hass and 17.3 μg/mL in Fuerte) than seed extracts, values similar to those reported for coffee extracts (5.1 μg/mL) [26], but higher than those found for Trolox standard (134 μg/mL) [27], caffeic acid (17 μg/mL), *p*-coumaric acid (74 μg/mL) [26], and strawberry guava peel and pulp extracts (32 and 18 μg/mL, respectively) [25]. However, all the extracts tested exhibited lower HOCl scavenging capacity compared with the standards used as references in this study. HOCl is an oxidant agent generated in neutrophils by the reaction of chloride ions (Cl^-^) with H_2_O_2_, catalyzed by myeloperoxidase. HOCl production is believed to constitute an important defense mechanism against microorganisms [28].

### Identification of phenolic compounds and quantification of free radical scavengers using HPLC-DAD coupled online with ABTS∙^+^

Five bioactive phenolic compounds, belonging to three different classes, were detected in peel and seed extracts of both avocado varieties and identified using HPLC-MS/MS: trans-5-*O-*caffeoyl-D-quinic acid (phenolic acid), catechin, epicatechin (flavan-3-ols) and procyanidins B_1_ and B_2_ (proanthocyanidins) (Table 2).

**Table 2:** Identification of phenolic compounds in peel and seed extracts of avocado varieties Hass and Fuerte using high performance liquid chromatography-tandem mass spectroscopy (HPLC-MS/MS) and quantification using high performance liquid chromatography (HPLC-DAD) coupled online with 2,2′-azinobis-(3-ethylbenzothiazoline-6-sulfonic acid) (ABTS)-based analysis.

Peak number^1^	Compound	RT (min)	Molecular ion [M-H]^-^ (m/z)	MS/MS ions (m/z)	Content (μg/mg)			
					Hass peel	Fuerte peel	Hass seed	Fuerte seed
1	Trans-5-*O-*caffeoyl-D-quinicacid	12.81	353	191	nd^2^	nd	1.63±0.03	5.74±0.25
2	Procyanidin B_1_	15.80	577	407 425 289	nd	nd	1.52±0.07	2.27±0.03
3	Catechin	18.70	289	245 205 179	nd	nd	3.64±0.03	8.13±0.38
4	Procyanidin B_2_	23.00	577	407 425 289	48.38±0.04	28.34±0.23	nd	nd
5	Epicatechin	26.28	289	245 205 179	40.21±0.24	30.40±0.28	10.27±0.08	11.06±0.03

^1^ Peak numbers refer to Fig 1.

^2^nd: not detected.

RT: retention time.

In peel extracts of Hass and Fuerte avocados, the following contents were found for the identified compounds: procyanidin B_2_−48.38 and 28.34 μg/mg, and epicatechin– 40.21 and 30.40 μg/mg, respectively (Table 2). These compounds have also been identified in peel extract of Hass avocado by others [6].

Catechin, epicatechin, procyanidin B_1_, and trans-5-*O-*caffeoyl-D-quinic acid were present in the seed extracts of both avocado varieties tested. Epicatechin was the compound found at higher concentrations in both varieties, Hass and Fuerte (10.27 and 11.06 μg/mg, respectively) (Table 2). Condensed tannins, phenolic acids, and flavonoids were the most representative groups of compounds found in avocado seeds in a previous study [5].

**Fig 1:**
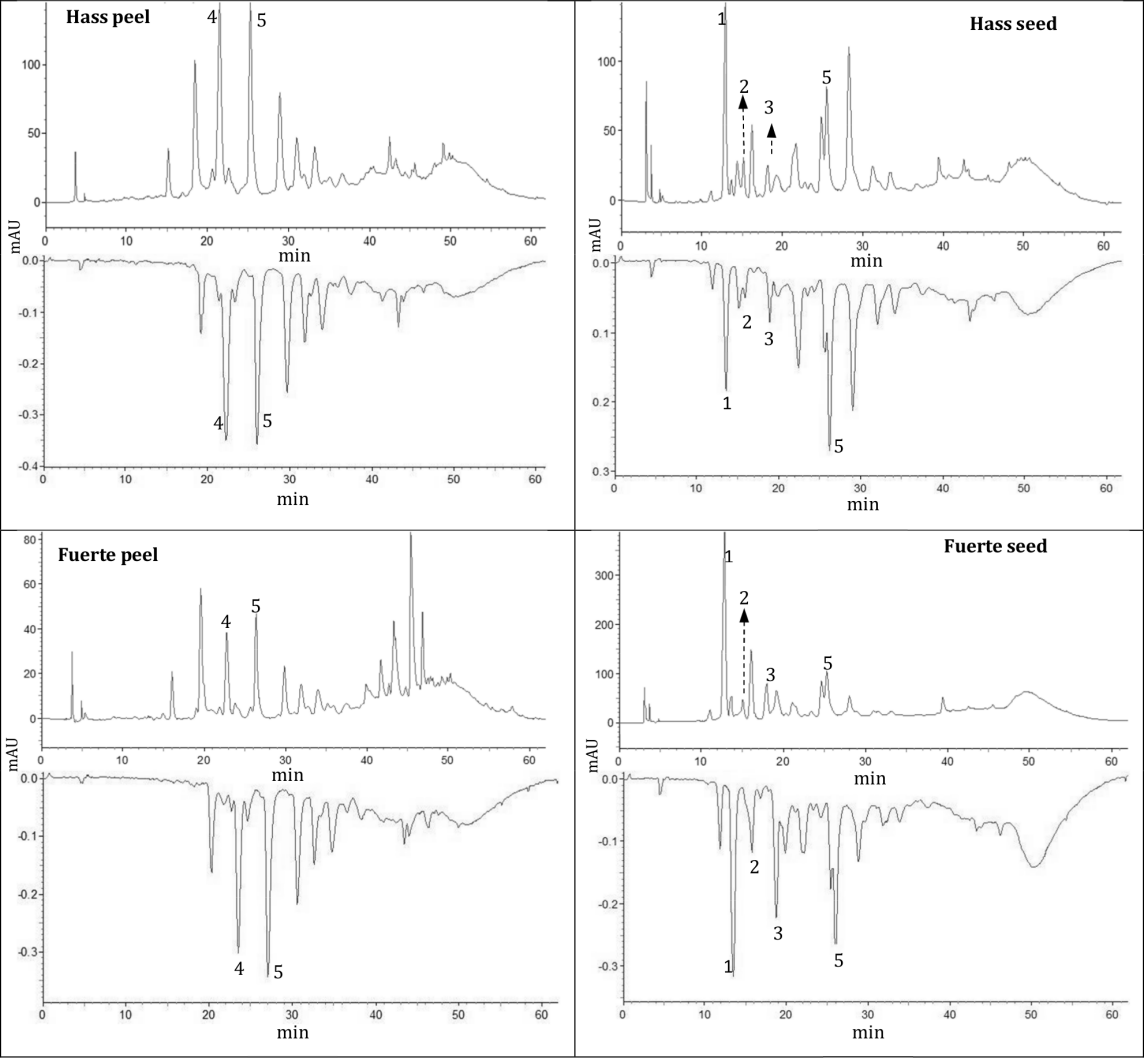
High performance liquid chromatography (HPLC) of peel and seed extracts of avocado varieties Hass and Fuerte detected at 280 nm (positive peaks–analytes separated by C18 column) and 734 nm (negative peaks–absorbance of postcolumn reaction of analytes with the ABTS). Peak numbers refer to Table 3.

The contents of procyanidin B_2_ and epicatechin in peel extracts of both varieties were responsible for antioxidant activities of 176.48, 75.32, 188.9, and 131.1 μmol/g Trolox, respectively, determined using HPLC-DAD coupled online with ABTS (Table 3). This is the first time these compounds have been shown to have a key role in total antioxidant activity in avocado agro-industrial by-products (Fig 1, Table 3).

**Table 3:** Determination of antioxidant activity of peel and seed extracts of avocado varieties Hass and Fuerte using high performance liquid chromatography (HPLC) coupled to diode array detector (DAD) and coupled online with 2,2′-azinobis-(3-ethylbenzothiazoline-6-sulfonic acid) (ABTS)-based analysis.

Avocado agro-industrial by-products	TEAC^1^ (μmol/g)				
	Trans-5-*O*-caffeoyl-D-quinicacid(peak 1)^2^	Procyanidin B_1_ (peak 2)	Catechin (peak 3)	Procyanidin B_2_ (peak 4)	Epicatechin (peak 5)
Hass peel	22.76	5.96	14.09	–	28.07
Hass seed	–	–	–	176.48	188.99
Fuerte peel	84.95	10.69	40.42	–	17.88
Fuerte seed	–	–	–	75.32	131.18

^1^TEAC: Trolox equivalent antioxidant capacity.

^2^Peak numbers refer to Figure.

In fact, a common characteristic of Hass and Fuerte peel and seed extracts was the presence of epicatechin. However, it should be noted that trans-5-*O-*caffeoyl-D-quinic acid, catechin, and procyanidin B_1_ were also important for the antioxidant activity in seed extracts of both varieties (Fig 1, Table 3).

### Cytotoxicity and anti-inflammatory activity

The cytotoxicity of different concentrations (0.1–100 μg/mL) of Hass and Fuerte peel and seed extracts was first examined using RAW 264.7 cells. None of the extracts tested exhibited cytotoxicity up to 10 μg/mL, and this concentration was then used in inflammatory *in vitro* assays.

The inflammatory process is a defense mechanism of an organism against invaders (microorganisms), and involves the participation of several chemical mediators capable of inducing vascular alterations with plasma protein extravasation and recruitment of defense cells [29]. Among the inflammation mediators are NO and the pro-inflammatory cytokines such as TNF-α [29, 30].

TNF-α is released by defense cells, mainly macrophages, and has a key role in the development of inflammation [31]. LPS present in bacteria have great capacity to stimulate TNF-α release by cells, and due to this, they are broadly employed in *in vitro* and *in vivo* anti-inflammatory studies [32, 33]. Among the pro-inflammatory activities of TNF-α, its capacity to induce the expression of cell adhesion molecules on endothelial cells stands out, because they are of paramount importance in the migration of defense cells to the inflammation site and in the destruction of the invader. Furthermore, TNF-α also controls the release of other pro-inflammatory and anti-inflammatory cytokines synthesized by defense cells [31].

NO, a colorless gas synthesized and released mainly by endothelial cells and macrophages, also plays a crucial role in the inflammatory process. The synthesis of NO by cells is regulated by the enzyme nitric oxide synthase (NOS) [30]. Among the major actions of NO in the inflammatory process are vasodilation and activation of pro-inflammatory and anti-inflammatory cytokines [30].

Despite being a defense mechanism, in a number of situations the inflammatory response may be exacerbated, in which case the defense cells of the immune system may attack the host. In this circumstance, it is necessary to use drugs capable of decreasing or inhibiting this event [29]. TNF-α and/or NO are ideal targets for anti-inflammatory drugs due to their role in the inflammatory process [30, 31].

**Fig 2:**
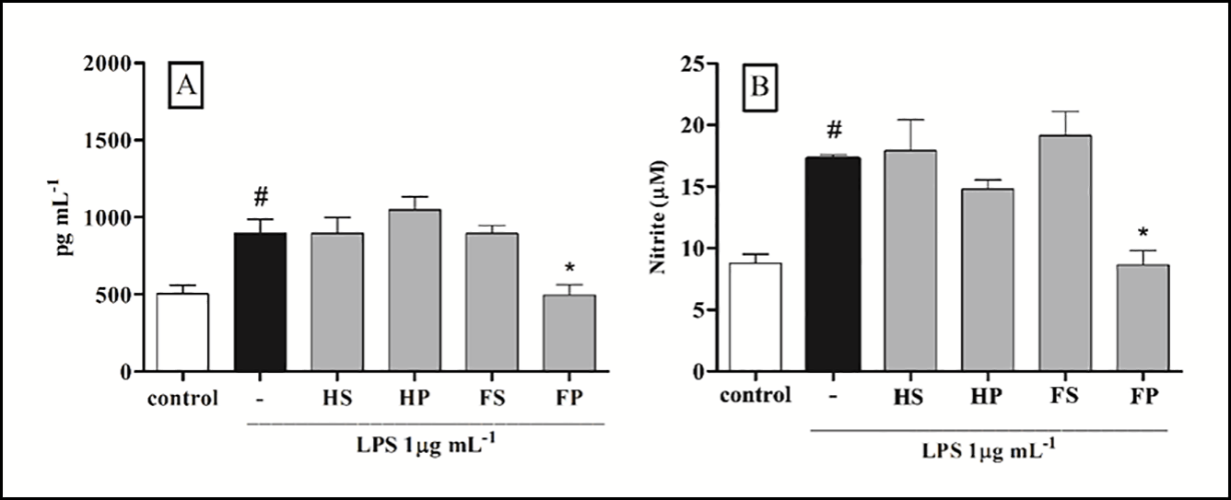
RAW 264.7 macrophages treated with peel and seed extracts of avocado Hass and Fuerte. (A) Levels of TNF-α in macrophages stimulated with LPS for 4 h. (B) Levels of nitrite in macrophages stimulated with LPS for 24 h. Cells incubated with the vehicle were used as control. Symbols indicate statistical difference (*p* < 0.05, Tukey’s test): # compared to control group; * compared to LPS group (-). HS: Hass seed; HP: Hass peel; FS: Fuerte seed; FP: Fuerte peel.

In the present study, the capacity of Hass and Fuerte peel and seed extracts to inhibit TNF-α and generate NO was assessed in LPS-stimulated RAW 264.7 macrophage culture. Based on our findings, Fuerte peel extract suppressed the release of TNF-α (495.3 pg/mL) (Fig 2A) and NO (8.5 μM) (Fig 2B) in activated RAW 264.7 macrophages (*p* < 0.05). These results suggest that the inflammatory activity of Fuerte peel extract may be related to the high contents of phenolic compounds found in its peel (Table 1). Studies show that this class of compounds is broadly known for its anti-inflammatory potential and inhibits the release of TNF-α and/or NO [32, 34].

Additionally, Fuerte peel extract exhibited high antioxidant activity, as indicated by the *in vitro* tests of ABTS, FRAP, and superoxide radical scavenging (Table 1). These findings also suggest that Fuerte peel extract may reduce the action of free radicals during the inflammatory process. Other studies have already demonstrated that free radicals are involved in the deleterious effects of the inflammatory process, and the consequent development of inflammatory diseases [35]. Induction of inflammation and tissue destruction are among the effects of free radicals [36]. Thus, based on these facts and on the present results, Fuerte peel extract can be considered a natural anti-inflammatory product, rich in bioactive phenolic compounds, capable of acting in different pathways of the inflammatory process such as the production of TNF-α and NO, as well as of inhibiting the action of free radicals.

## Conclusions

In conclusion, this study indicated that the use of ethanol for the obtention of Hass and Fuerte peel and seed extracts resulted in a product with high content of bioactive phenolic compounds such as catechin, epicatechin, procyanidins B_1_ and B_2_, and trans-5-*O*-caffeoyl-D-quinic acid. High antioxidant activity as well as suppression of TNF-α and NO generation properties were found in Fuerte peel extract. Considering that the production of avocado varieties Hass and Fuerte has been expanding in response to the increased consumption of this fruit worldwide, these findings allow the conclusion that avocado agro-industrial by-products are important sources of natural antioxidant and anti-inflammatory agents that can be employed in several industries. Moreover, extracts obtained by green extraction can be used as ingredients in the formulation of functional foods.
